# Linking Electrostatic-Induced Chain Stiffening to Heat Flow in Amorphous Polymers

**DOI:** 10.1021/acsmacrolett.6c00120

**Published:** 2026-04-15

**Authors:** Debashish Mukherji, Marcus Müller

**Affiliations:** Institut für Theoretische Physik, 9375 Georg−August−Universität Göttingen, 37077 Göttingen, Germany

## Abstract

Amorphous polymers are soft materials whose thermal properties are dominated by weak, nonbonded interactions that strongly suppress heat flow, limiting the thermal transport coefficient, κ, to below 0.40 W m^–1^ K^–1^. Recent experiments, however, show that electrostatic modification of poly­(acrylic acid) (PAA) can increase κ beyond 1.00 W m^–1^ K^–1^ in highly ionized systems, compared to κ ≃ 0.33 W m^–1^ K^–1^ for uncharged PAA. Using molecular-dynamics simulations of a bead–spring polymer model, we study the effect of ionization on κ in amorphous polymers. In agreement with experiment, we observe a more than 2.5-fold enhancement in κ, driven by electrostatically induced, local chain stiffening that increases the bonded contribution to κ. These results identify a generic mechanism for tuning κ across a broad class of charged polymers.

## Graphical abstract

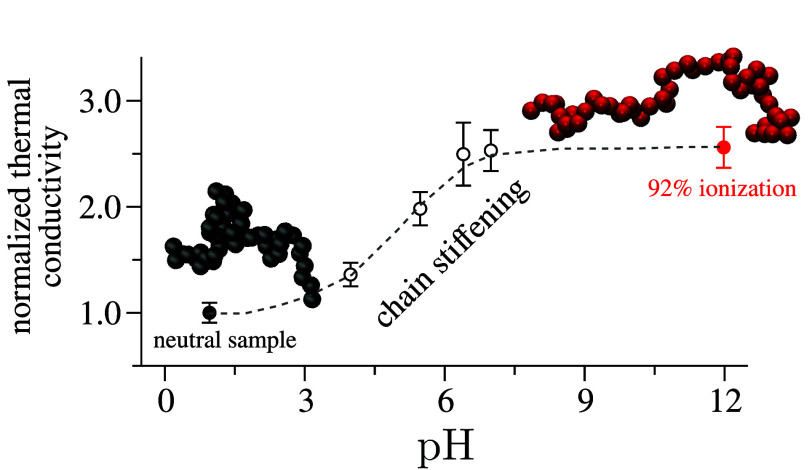

The thermal transport coefficient, κ, is a fundamental transport property governing heat dissipation in materials.^1–3^ Despite the broad utility of polymers in applications ranging from thermal insulation to flexible electronics and energy storage,^4–6^ most polymers exhibit intrinsically low κ compared to crystalline solids.^3^ This limitation is particularly pronounced in amorphous polymers, where the lack of long-range order and the dominance of weak nonbonded interactions lead to strong vibrational scattering and short mean free paths, Λ, of phonons, yielding typical κ values of 0.1–0.4 W m^–1^ K^–1^.^7–11^

Generally, κ in polymers is dictated by a delicate balance between fast intrachain heat transfer along (stiff) backbone bonds and slow intermolecular energy transfer^12,13^ mediated by soft van-der-Waals (vdW),^8,14^ hydrogen-bonding (H-bond),^8,15^ or electrostatic interactions.^16,17^ As a result, κ is highly sensitive to chain conformation, local packing, and intermolecular interactions.^7,9,10,18^ Considerable effort has therefore been devoted to enhancing polymer thermal conductivity through macromolecular design, including polymer blending,^8,19^ chain alignment,^20–22^ filler incorporation,^4,5^ and modification of ionization.^16,17^

Recent experiments have demonstrated that electrostatic modifications can substantially enhance κ even in fully amorphous polymers.^16^ In particular, increasing the degree of ionization, α, in poly­(acrylic acid) (PAA) from the neutral state to ∼92% raises κ from 0.33 W m^–1^ K^–1^ ^8^ to nearly 1.00 W m^–1^ K^–1^.^16^ Charged polymer systems exhibit behavior distinct from neutral melts due to long-range electrostatic interactions, which strongly couple chain conformation, molecular packing, and transport properties.^23–25^ While these interactions primarily affect local structure, their implications for the κ-behavior remain poorly understood at the microscopic level.

In this work, we systematically vary the degree of ionization, α to (1) quantify its influence on κ and to disentangle the microscopic contributions governing macroscopic κ, (2) correlate κ with chain conformations and local stiffening, (3) identify the microscopic mechanisms responsible for enhanced thermal transport in amorphous charged polymers, and (4) provide a general understanding of heat-transport enhancement in charged polymer systems.

To accomplish these objectives, we investigate the universal aspects of electrostatic enhancement of κ within the framework of a chemically nonspecific bead–spring polymer model.^26^ Our simulations are performed using the LAMMPS molecular dynamics package.^27^ All quantities are reported in Lennard-Jones (LJ) reduced units, with length σ and energy ϵ. These choices define the fundamental unit of time as $\tau =\sigma \sqrt{m/\epsilon }$ , where *m* is the monomer mass. κ is computed using the equilibrium Green–Kubo formalism (see Supplementary Section S3) in the microcanonical ensemble.

Each sample contains *N*_c_ polymer chains, each composed of $N_{l}=50$ monomers. The total number of particles *N* includes both monomers and counterions. Six different samples are prepared with varying degree of ionization, α (see Supplementary Table S1). Monomers and counterions are assigned the same effective size in the simulations (see Supplementary Section S1). Model-specific details—including system sizes, interaction potentials, sample preparation, and κ calculations—are provided in Supplementary Sections S1, S2, and S3.

We begin by presenting the central result of our study in Figure 1(a), where κ is plotted as a function of pH value (or equivalently, α), represented by the ○ symbols. Two prominent features are apparent and are in good agreement with experimental data for ionized poly­(acrylic acid) (PAA), shown by red △ symbols:^16^ (i) a sharp increase in κ occurs around pH ≃ 6.0 (corresponding to α ≃ 50%), and (ii) the overall magnitude of κ increases by nearly a factor of 2.5 over the full range of pH values studied.

**Figure 1:**
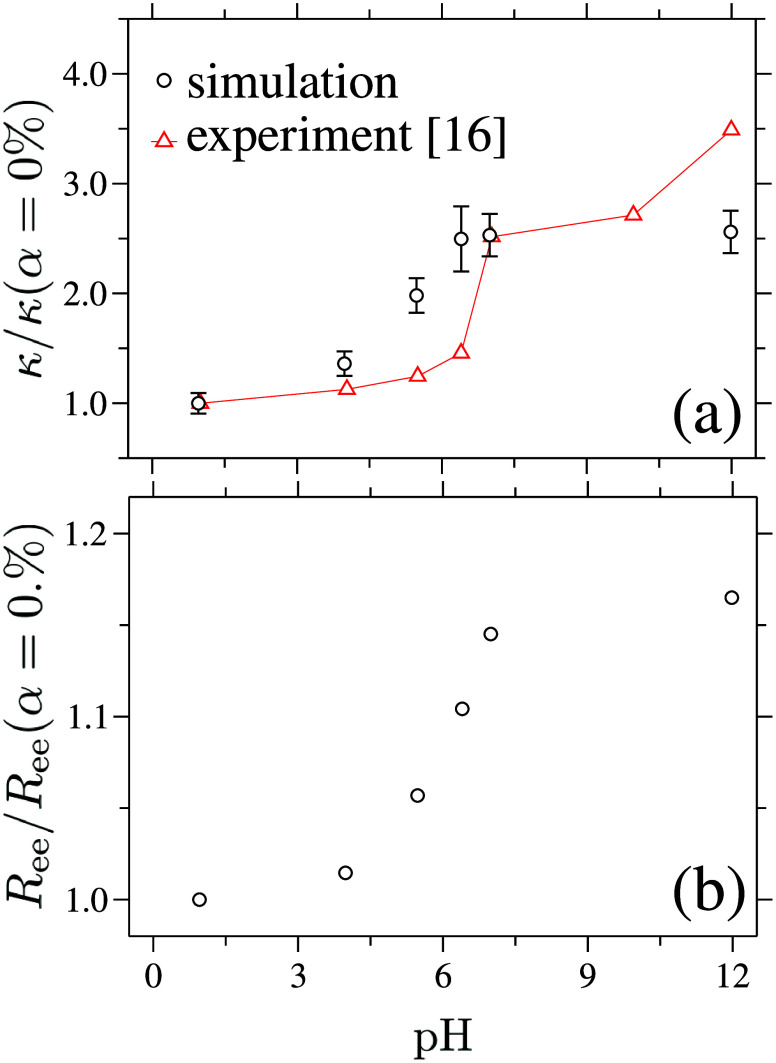
(a) Thermal conductivity κ as a function of pH (or equivalently, the degree of ionization α). The data are normalized by the thermal conductivity of the uncharged system, κ­(α = 0%) = 4.52 ± 0.42*k*_B_τ^–1^σ^–1^. Error bars represent the standard deviation obtained from 100 independent calculations. For comparison, experimental data for ionized poly­(acrylic acid) (PAA)^16^ are included and normalized by κ = 0.33 W m^–1^ K^–1^ for uncharged PAA. (b) Chain end-to-end distance *R*_ee_ as a function of pH, normalized by the neutral value *R*_ee_(α = 0%) = 8.32σ. Error bars for *R*_ee_ are smaller than the symbol size.

It is well established^23–25^ that increasing the fraction of charged monomers along a polymer backbone enhances electrostatic repulsion between like charges, driving the chains toward more extended conformations. In this context, the sharp increase in κ observed near pH ≃ 6.0 (corresponding to α ≥ 50%), as well as the overall scaling with pH, point to α-induced conformational changes of the polymer chains. This behavior is clearly reflected in the increase of the end-to-end distance *R*_ee_ shown in Figure 1(b), indicating that the polymer chains become progressively extended relative to the neutral system (α = 0%). Note that for these systems we find (*R*_ee_/*R*_g_)^2^ ≈ 6.0, indicating that on longer scales the chain configurations obey Gaussian-like statistics. However, we wish to highlight that conformation statistics on length scales larger than the characteristic intramolecular-transport length along the polymer backbone do not contribute to κ.

To gain a more detailed understanding of chain conformations across different length scales, we examine the single-chain structure factor, using $S\left(k\right)=N_{l}^{-1}\left\langle \right|\overset{N_{l}}{\underset{j=1}{\sum }}e^{ik\cdot r_{j}}{|}^{2}\rangle$ , where *r*_*j*_ is the position of the *j* ^th^ segment, **k** the wave vector, and ⟨...⟩ denotes averaging over all chains within a sample and 100 independent samples. Representative *S*(*k*) data for two different α values are shown in Figure 2. Over a range of wavenumbers, 2.0 ≤ *kR*_g_ ≤ 10.0 for α = 0% and 2.0 ≤ *kR*_g_ ≤ 7.0 for α = 92%, *S*(*k*) ∼ *k* ^–2^, characteristic of random-walk (Gaussian) chain conformations. This Gaussian scaling persists across all α values (data not shown).

**Figure 2:**
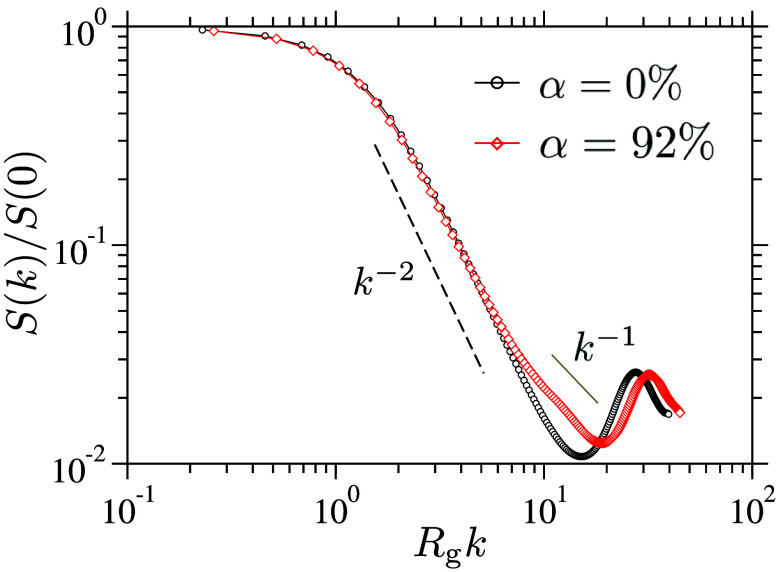
Static structure factor, *S*(*k*), is shown for two different degrees of ionization, α. At intermediate wavevectors, a power law decay, *S*(*k*) ∼ *k* ^–2^, indicates that the chains adopt a random-walk (Gaussian) conformation. For fully ionized chains (α = 92%), a rod-like scaling, *S*(*k*) ∼ *k* ^–1^, is observed at short length scales, corresponding to distances below approximately 3.75σ. This behavior reflects the strong electrostatic extension of the chain segments, which suppresses local coiling and results in more aligned, stiffened chain segments at these length scales. The ordinate is normalized by the chain length, $S\left(0\right)=N_{l}=50$ , while the abscissa is scaled by the single-chain radius of gyration, *R*_g_. For the neutral (α = 0%) and ionized (α = 92%) systems, the corresponding radii of gyration are *R*_g_ = 3.65σ and 4.19σ, respectively.

In contrast, at high ionization levels—for example, at α ≃ 92%—a clear rod-like scaling, *S*(*k*) ∼ *k* ^–1^, emerges over the range 7.0 ≤ *kR*_g_ ≤ 15.0. This behavior originates from strong, intrachain electrostatic repulsions between neighboring, charged monomers, which dominate at short length scales where electrostatic screening is ineffective. These repulsive interactions extend the polymer backbone, suppress local coiling, and substantially reduce conformational fluctuations. As a result, the chains exhibit local bending stiffness over a characteristic length scale, l≃3.75σ , estimated from the crossover between the *k* ^–2^ and *k* ^–1^ regimes in the α = 92% data shown in Figure 2.

The existence of these two regimes—rod-like behavior at short length scales and Gaussian statistics at larger scales—reveals a hierarchical organization of chain conformations in ionized polymers. This multiscale structural organization is central to understanding how microscopic conformational changes propagate across length scales and ultimately influence the macroscopic κ. In this context, it is important to recall that κ is governed by three key microscopic quantities: (i) the mean free path of vibrational excitations, Λ, (ii) the sound velocity, *v*, and (iii) the volumetric specific heat, *c*. Ionization alters local chain conformation, intermolecular interactions, and packing. Thereby, it affects each of these contributions individually, leading to measurable changes in κ. In the following, we will look into each contribution in turn.

(i) Local chain stiffening enhances the efficiency of energy propagation along covalently bonded backbone segments by reducing scattering of vibrational modes and thus increasing Λ. Since the energy-transfer rate between two bonded monomers is 50–100 times faster than that between two nonbonded monomers,^12,13^ chain stiffening naturally leads to faster heat flow along the chain.^20–22^

We quantify the separate bonded, κ_b_, and nonbonded, κ_nb_, contributions to the total κ in Figure 3(a). Both κ_b_ and κ_nb_ increase with the pH value; however, κ_b_ exhibits a markedly sharper transition, increasing by nearly a factor of 5 for pH ≥ 6.0 (or α ≥ 50%) relative to the neutral system (see ○ symbols in Figure 3(a)). In contrast, κ_nb_ increases only modestly with pH (see ● data set in Figure 3(a)). Nevertheless, κ_nb_ dominates for pH ≤ 6.0 (or α ≤ 50%), while the bonded contribution, κ_b_, only becomes dominant at higher pH, accounting for more than 50% of the total κ (see ○ symbols in Figure 3(b)). This crossover is further illustrated in Figure 3(b), which shows the fractional contributions to the total κ.

**Figure 3:**
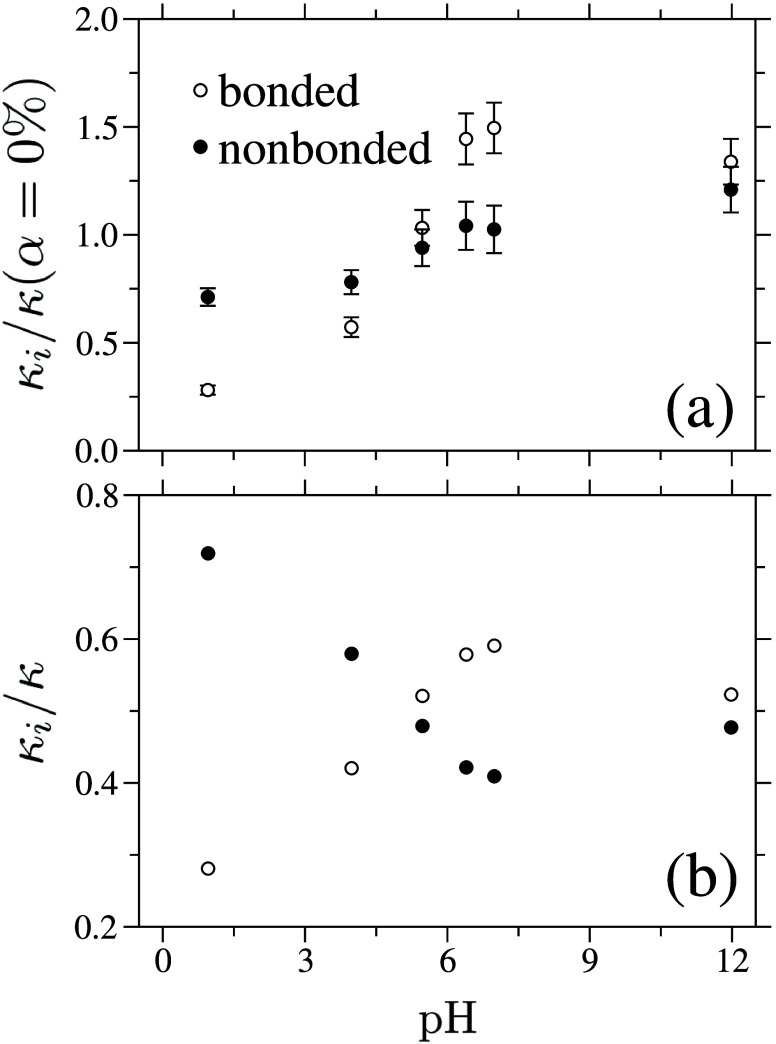
(a) Bonded, κ_b_, and nonbonded, κ_nb_, contributions to the thermal conductivity, κ, as a function of pH. All data are normalized by the bonded thermal conductivity of the uncharged system, κ­(α = 0%) = 4.52 ± 0.42*k*_B_τ^–1^σ^–1^. (b) pH dependence of the fractional contributions of the bonded and nonbonded components to the total thermal conductivity, κ_b_/κ and κ_nb_/κ, respectively.

The above behavior demonstrates that ionization-induced chain extension strongly enhances intrachain heat transport. Reduced conformational bends and kinks along the polymer backbone promote more efficient backbone vibrations and enable coherent vibrational energy transfer along covalent bonds.^22^ These results indicate that structural reorganization predominantly amplifies κ_b_, consistent with previous observations for phase-separated thermoresponsive polymers in solution.^28,29^ We also prepared an additional test sample at α = 84% to further examine the effect of chain extension on κ; details are provided in Supplementary Section S4.

It should be noted that ionization introduces counterions, which dilute the density of bonded interactions and would reduce κ_b_ if chain stiffness remained unchanged. The opposite trend observed in Figure 3(a) thus underscores the dominant role of chain stiffening in enhancing κ.

(ii) In addition to chain extension and efficient heat transport along the backbone of individual chains, elastic wave propagation through the sample—mediated by a combination of bonded and nonbonded interactions—also plays an important role. Therefore, we computed the longitudinal elastic constant *C*_11_ for both the uncharged system and the ionized system with α = 92%, using our recently developed method.^30^ We find that *C*_11_(α = 92%)/*C*_11_(α = 0%) ≃ 1.97. Since the longitudinal sound velocity scales as $v\propto \sqrt{C_{11}}$ , this increase in stiffness corresponds to *v*(α = 92%)/*v*(α = 0%) ≃ 1.41. Consequently, material stiffness gives an additional contribution of approximately 40% enhancement in total κ.

Note that the enhancement in *C*_11_ from low ionization (pH ≤ 6.0) to high ionization (pH ≥ 6.0) is approximately 1.97 for all samples. It is important to emphasize that this increase in *C*_11_ arises from the combined effects of chain stiffening and electrostatic interactions. While local chain stiffness contributes to *C*_11_, our additional simulations—where chain stiffening is artificially enhanced (see Supplementary Section S4A)—demonstrate that *C*_11_ does not necessarily increase proportionally. In other words, the local bending stiffness of individual chains and the bulk stiffness are correlated, but not directly proportional. A direct comparison of the variation of *C*_11_ and κ with *R*_ee_ is presented in Supplementary Figure S4.

Overall, mechanisms (i) and (ii) provide the dominant contributions to κ and together account for a 2.15-fold increase for the highly ionized polymer relative to its neutral counterpart. This combined enhancement underscores the central role of electrostatically induced chain straightening and bonded heat-transfer pathways in governing heat flow in ionized amorphous polymers.

(iii) Finally, we compute the vibrational density of states, *g*(ν) = ∫_0_^*∞*^ cos­(2*πνt*)­ψ­(*t*)/ψ­(0) d*t*, from the Fourier transform of the mass-weighted velocity autocorrelation function ψ­(*t*) = ∑_*i*_ *m*_*i*_⟨**V**_*i*_(*t*)·**V**_*i*_(0)⟩, following refs 31 and 32 . Representative spectra for two α are shown in Figure 4. With increasing α, the vibrational peaks shift to higher frequencies, reflecting progressive stiffening of the system.

**Figure 4:**
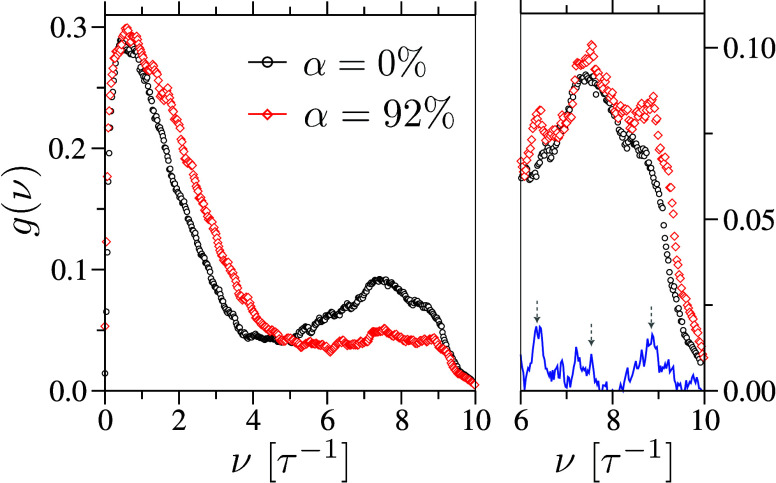
Left panel: vibrational density of states, *g*(ν), for two different degrees of ionization, α. The right displays the same data, but with the α = 92% spectrum scaled by the number of monomers in the sample for better comparison. With increasing α, the polymer concentration decreases due to the presence of counterions. The blue curve in the inset represents the difference between the two spectra. Arrows mark the characteristic frequencies associated with (left) librational motion at ν ≃ 9.0τ^–1^ and (right) flexural vibrations at ν ≃ 7.5τ^–1^, and the FENE bond at ν ≃ 6.5τ^–1^.

The apparent weakening of the peak near ν ≃ 7.0 τ^–1^ at α = 92% arises from dilution of the polymer vibrational signal by counterions. Upon normalizing *g*(ν) by the number of polymer segments (right panel of Figure 4), the vibrational features become more pronounced, and the difference spectrum clearly resolves librational (ν ≃ 9.0 τ^–1^), flexural (ν ≃ 7.5 τ^–1^), and bond-stretching (ν ≃ 6.5 τ^–1^) modes.^33^ Moreover, increasing α leads to systematically narrower peaks, indicating longer vibrational lifetimes and reduced anharmonic damping. To quantify the contribution of *g*(ν) to κ via the volumetric specific heat *c*, we estimate an approximately 10% increase for α = 92% relative to the neutral polymer when applying (classical) Boltzmann weighting.

In summary, we have used molecular-dynamics simulations of a bead–spring polymer model to investigate heat transport in dense, solvent-free charged polymers with counterions, focusing on how the degree of ionization, α, modulates the thermal transport coefficient, κ. Our results reveal a pronounced enhancement of κ with increasing α, in good agreement with experiments on ionized poly­(acrylic acid).^16^ In particular, we observe a sharp increase near pH ≃ 6.0, corresponding to α ≃ 50%. This enhancement arises from a subtle interplay between α-induced chain extension, increased material stiffness, and modifications of the vibrational spectrum, rather than simply from the presence of charges or counterions. Quantitatively, the total increase in κ can be decomposed into a factor of 1.5 due to local chain stiffening, a factor of 1.4 associated with increased sound velocity arising from enhanced stiffness, and a remaining factor of approximately 1.1 originating from changes in the heat capacity. Together, these contributions account for the observed ≃2.5-fold increase (see Figure 1(a)) in κ between the neutral polymer and the highly charged polyelectrolyte.

Since chain stiffening is a primary factor in enhancing κ, polymers composed of multiple short blocks with different pH-responsive ionization thresholds could achieve greater tunability. In such block copolymers, highly ionized blocks stretch while less ionized blocks remain relatively coiled, allowing systematic control of κ by adjusting block sizes. A potential example is derivatives of regenerated cellulose: pure cellulose is nearly nonionizable (pH ∼ 12–13), whereas carboxymethyl cellulose (CMC) is highly ionizable (pH ∼ 4.3–4.5). Combining these blocks could yield polymers with tunable chain extension and κ.

Further studies are needed to develop a macromolecular toolbox for tuning κ in amorphous polymers. Our findings demonstrate that α or environmental factors such as pH serve as powerful, reversible knobs for controlling κ, offering new design strategies for polyelectrolytes and charged polymer networks with potential applications in thermal management, energy storage, and adaptive materials.

## Supplementary Material



## Data Availability

The scripts and the data associated with this research are available upon reasonable request from the corresponding author.

## Notes

Detailed description of the generic model, simulation methodology, sample preparation protocol, thermal transport calculations, and some additional data for chain stiffening (PDF)
