# Supplementary material for: Linking Electrostatic-Induced Chain Stiffening to Heat Flow in Amorphous Polymers
Source: ACS Macro Lett. 2026 Apr 15;15(5):685–9. doi: 10.1021/acsmacrolett.6c00120 (PMC13192311; doi:10.1021/acsmacrolett.6c00120)
Supplement: Supplementary file 1 [file mz6c00120_si_001.pdf]

Supporting information: "Linking Electrostatic–Induced Chain Stiffening to Heat Flow in Amorphous Polymers"

Debashish Mukherji* and Marcus Müller
Institut für Theoretische Physik, Georg–August–Universität Göttingen, 37077 Göttingen, Germany

In this supplementary document, we provide additional supporting data to strengthen some of the claims presented in the Main text.

S1. POLYMER MODEL AND INTERACTIONS

We employ a generic molecular-dynamics simulation framework based on the bead-spring polymer model [1] using the LAMMPS molecular-dynamics package [2]. In this model, all monomers interact via a purely repulsive Lennard–Jones (LJ) potential of the 6-12 form,

$$U_{\rm LJ}(r) = \begin{cases} 4\epsilon\left[\left(\frac{\sigma}{r}\right)^{12} - \left(\frac{\sigma}{r}\right)^{6}\right] + \epsilon, & r \le r_c, \\ 0, & r > r_c, \end{cases} \quad (1)$$

where the cutoff distance is set to $r_c = 2^{1/6}\,\sigma$. The LJ energy scale ϵ and length scale σ are taken to be identical for all particle pairs, including polymer monomers and counterions. This choice of taking the same σ for both – monomer and counterion – is motivated by the OPLS–AA estimate of $\sigma \simeq 0.33$ nm [3] for Na^+, which is also the typical size of an acrylic acid monomer, approximately 0.35 nm [4].

Adjacent monomers along each polymer chain are additionally connected by a finitely extensible nonlinear elastic (FENE) bonding potential,

$$U_{\rm FENE}(r) = -\frac{1}{2}kR_0^2 \ln\left[1 - \left(\frac{r}{R_0}\right)^2\right], \quad (2)$$

with a maximum bond extension $R_0 = 1.5\,\sigma$ and spring constant $k = 30\,\epsilon/\sigma^2$.

The equations of motion are integrated using the velocity-Verlet algorithm with a time step of $0.01\,\tau$, which ensures numerical stability and accurate energy conservation. The system temperature is maintained at $T = 1.0\,\epsilon/k_{\rm B}$ by means of a Langevin thermostat with a damping coefficient $\gamma = 1\,\tau^{-1}$.

Long–range electrostatic interactions are treated using the particle–particle particle–mesh (PPPM) method. The PPPM solver is employed with a target relative force accuracy of 10^{-4}, ensuring well–converged electrostatic energies and forces while maintaining computational efficiency.

*debashish.mukherji@theorie.physik.uni-goettingen.de

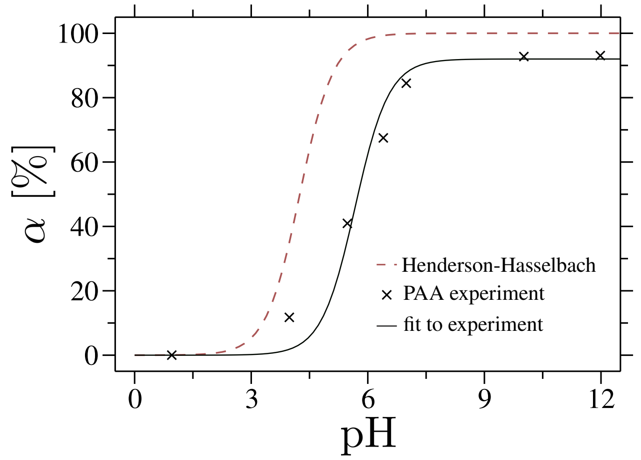

FIG. S1: Degree of ionization, α, as a function of pH for amorphous poly(acrylic acid) (PAA). Experimental values of α obtained from Fourier–transform infrared spectroscopy are taken from Ref. [5]. The solid line shows a fit to the experimental data, using $\alpha = 10^{\rm pH-pK_A}/\left(1 + 10^{\rm pH-pK_A}\right)$, yielding an effective $pK_{\rm A} = 5.67$, though the maximum α in the experiment is about 92%. For comparison, the Henderson–Hasselbalch prediction is also shown using $pK_{\rm A} = 4.25$ for the acrylic acid monomer.

We note in passing that electrostatic interactions between bonded monomers are excluded. Therefore, the presence of charges does not affect the bond length, which remains $\ell_{\rm b} \simeq 0.97\,\sigma$ [1] across all samples, independent of degree of ionization, α.

S2. SAMPLE PREPARATION

To prepare charged samples at different α, we employ a simple protocol motivated by the experimentally measured α values at various pH conditions [5], rather than implementing a dynamic constant-pH ionization scheme [6].

Starting from an equilibrated neutral sample (i.e., $\alpha = 0\%$) consisting of $N_{\rm c} = 1000$ chains (corresponding to $N = 5 \times 10^4$ particles) at a particle number density $\rho_N = 0.85\,\sigma^{-3}$, charged samples are generated as follows: (i) A subset of chains is first selected such that the total number of monomers and counterions remains $N \simeq 5 \times 10^4$; details of the system sizes are provided in Table S1. Once the chains are selected, the FENE bonds between the remaining chains are removed, producing a sample of individual chains and monomers. This system is equilibrated using the model described in Section S1

for a time $t = 10^5\,\tau$ to ensure complete miscibility.
(ii) Within the selected chains, a fraction α of monomers is randomly assigned a unit negative charge to produce systems with varying degrees of ionization. The charged monomers are distributed along each polymer backbone and across all chains, ensuring that no two chains share the same sequence of charged and neutral monomers. The selected values of α correspond to those investigated experimentally in Ref. [5] and are highlighted by the $\times$ data set in Figure S1. For each value of α, 100 independent samples are prepared for property calculations, which allows us to have better averaging and closely mimic the experimental systems.
(iii) Charge neutrality is ensured by introducing an equal number of monovalent counterions as the charged monomer into each sample, chosen from the remaining monomers without bonds. A few unassigned particles (neither monomer of a polymer nor counterions) are removed.

Each ionized monomer carries a unit negative charge and monovalent counterions carry unit positive charge. We note that the experimental study also reported counterion condensation, with Na^+ ions preferentially accumulating near the negatively charged acrylic acid monomers [5]. A summary of the system compositions, including the number of charged monomers for each value of α, is provided in Table S1.

TABLE S1: System sizes and compositions as a function of the degree of ionization α (or pH) [5], showing the total particle number N, polymer chain count N_c, and number of ionized monomers N_{ionized}.

pH	α [%]	N	N_c	N_{ionized}
0.95	0	50000	1000	0
3.98	12	49280	880	5280
5.47	40	49000	700	14000
6.39	67	49766	596	19966
6.99	84	49680	540	22680
11.98	92	49920	520	23920

Each simulated sample consists of N_c polymer chains, each composed of $N_\ell = 50$ monomers. In all cases, the simulation box contains a total of N particles, including polymer monomers and counterions (see the third column of Table S1). This system size is chosen to ensure adequate statistical sampling while minimizing finite-size effects.

Note that an uncharged polymer sample typically exhibit a pressure of $p \simeq 5.0\,\epsilon/\sigma^3$ at a total number density of $\rho_N \simeq 0.85\,\sigma^{-3}$. Accordingly, all samples were equilibrated at this pressure. The density equilibration for each sample was carried out for 10^5 MD steps, corresponding to a simulation time of $t = 10^3\,\tau$. Subsequently, each sample with all α is further equilibrated for at least 6×10^6 molecular dynamics (MD) steps under the canonical ensemble, corresponding to a simulation time of $t = 6 \times 10^4\,\tau$. This equilibration time exceeds by more than an order of magnitude the characteristic relaxation time required for a polymer chain to diffuse a distance comparable to its end–to–end length, R_{ee}. As a result, the systems are well equilibrated prior to data collection. Representative mean–squared displacement, $g(t)$, data for two representative samples are shown in Figure S2. Note that we have $g(t)$ for all α, however, for clarity of data presentation, we only show two cases.

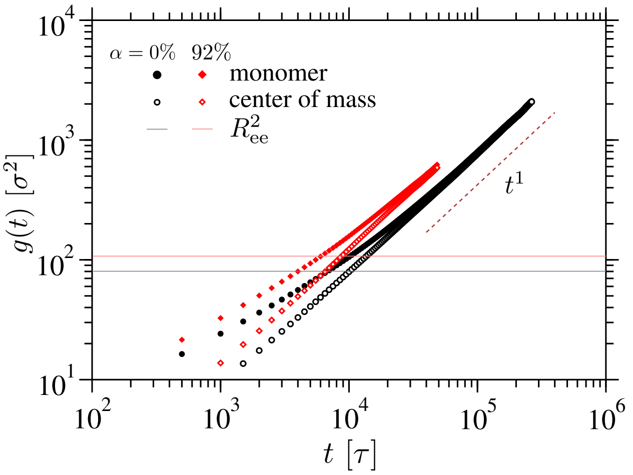

FIG. S2: Mean–squared displacement $g(t)$ of monomers (empty symbols) and center–of–mass (solid symbols) as a function of time t for polymer samples with two degrees of ionization, α, i.e., two limits of fully uncharged and a fully charged samples. The dashed line indicates the diffusive regime where $g(t) \sim t$.

The equilibrated samples obtained after the procedure described above are subsequently employed for the calculation of both structural and transport properties. These properties include, but are not limited to, R_{ee}, single chain structure factor, $S(k)$, and thermal transport coefficient, κ.

S3. CALCULATION OF κ

The thermal transport coefficient, κ, is computed using the Green-Kubo formalism [7]. All calculations are performed in the microcanonical (NVE) ensemble, which ensures energy conservation and avoids artifacts from thermostats during the evaluation of heat flux correlations. The heat flux auto-correlation function (HFACF) is defined as,

$$\phi(t) = \langle \mathbf{J}(t) \cdot \mathbf{J}(0) \rangle, \tag{3}$$

where $\mathbf{J}(t)$ is the instantaneous heat flux vector at time t. The HFACF is sampled with a time step of $0.01\,\tau$ over the interval $0 \le t \le 10\,\tau$, which is at least an order of magnitude longer than the characteristic decorrelation time of the heat flux. During a total simulation of $10^3\,\tau$,

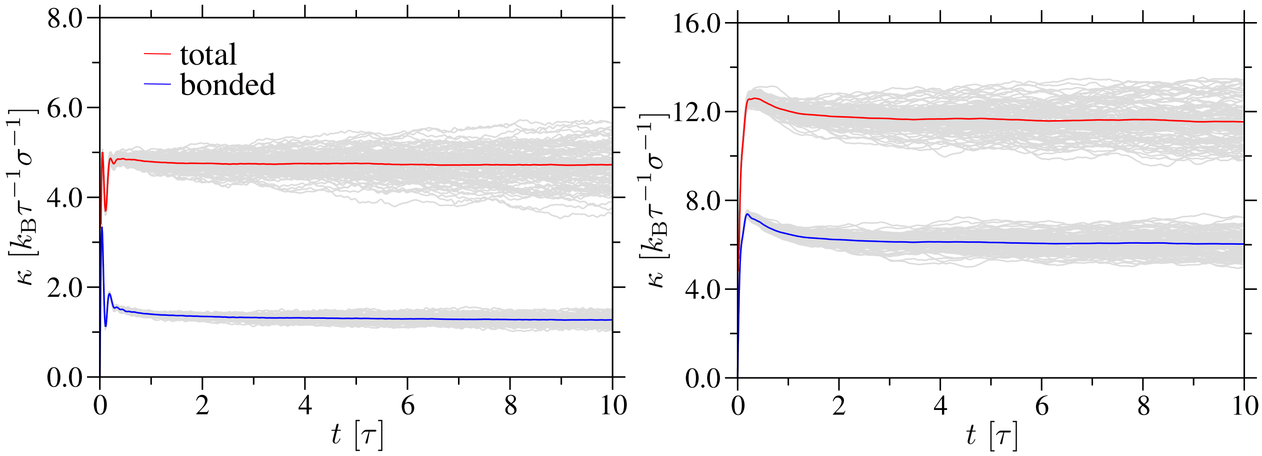

FIG. S3: Cumulative thermal conductivity κ for two degrees of ionization, α. The data are computed using the Green–Kubo relation [Eq. 4] for an uncharged polymer melt at α = 0% (left panel) and for a highly ionized system at α = 92% (right panel). The plateau of the time integral at long times ($t \gtrsim 8\,\tau$) is used to extract the final values of κ. For each sample, 100 independent κ calculations are performed using stochastically independent configurations (gray lines).

the HFACF is accumulated and a running average is computed to improve statistical reliability.

The thermal conductivity is obtained via the Green-Kubo integral,

$$\kappa = \frac{v}{3k_{\rm B}T^2}\int_0^{\mathcal{T}} \phi(t)\, dt, \tag{4}$$

where v is the system volume, T is the temperature, and $k_{\rm B}$ is the Boltzmann constant. In practice, the tail of $\phi(t)$ may exhibit slow convergence due to finite system size and limited simulation time. To address this, κ is calculated from the plateau region of the cumulative integral, averaging over times $t \geq 8\,\tau$ (see representative data in Figure S3).

We note in passing that $\mathbf{J}$ is computed as a combination of kinetic and potential contributions. The potential contribution can be further separated into bonded and non-bonded (pair) interactions, based on the per-atom potential energy and forces. This allows one to analyze how different types of interactions contribute to the overall κ. For this purpose, the heat flux can be computed selectively by including only non-bonded interactions using the 'pair' option or only bonded interactions using the 'bond' option in the `compute heat/flux` command in LAMMPS [2]. For each sample, we calculate κ, in three distinct ways: (a) Total κ, including all interactions, (b) κ considering only non-bonded pair interactions, and (c) κ considering only bonded interactions. This decomposition provides insight into the relative contributions of chain connectivity and non-bonded interactions to the overall enhancement of κ in the polymer system.

S4. EFFECT OF STIFFENING ON κ

In the Main Figure 1, we present the variation of $R_{\rm ee}$, κ, and C_{11} as functions of pH (or, equivalently, the degree of ionization α). To further clarify the interdependence of these quantities, we present here a unified representation in Figure S4 that directly correlates them. As $R_{\rm ee}$ increases, both κ and C_{11} increase systematically, highlighting the role of chain stiffening in governing both thermal transport and mechanical response. However, while local chain stiffness contributes to C_{11}, the relationship is not strictly one-to-one, indicating that bulk stiffness is influenced by additional factors such as collective packing and electrostatic interactions.

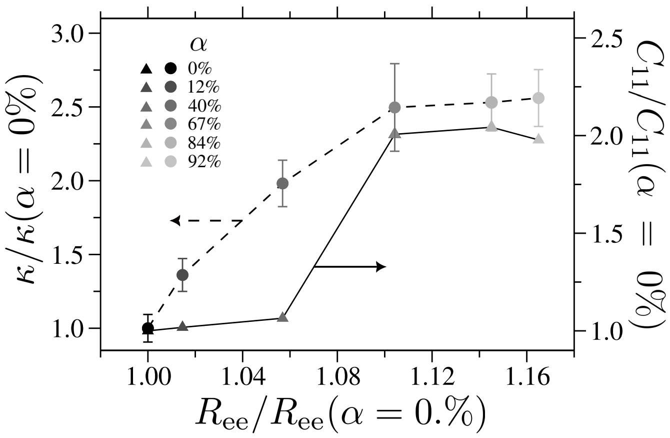

FIG. S4: Variation of thermal conductivity, κ (left axis), and elastic modulus, C_{11} (right axis), as a function of end-to-end distance, $R_{\rm ee}$. All quantities are normalized by their respective values for the neutral sample (α = 0%), for which $C_{11} = 12.23\,\epsilon/\sigma^3$, $\kappa = 4.52 \pm 0.2\, k_{\rm B}\tau^{-1}\sigma^{-1}$, and $R_{\rm ee} = 8.32\,\sigma$. The corresponding α values for each data point are also indicated (see Table S1). Arrows indicate at the corresponding y–axes.

To further investigate the dominant role of chain stiffening in enhancing thermal transport, we examined an additional sample at α = 84%. For this sample, we

started from the configuration used for the calculation of κ in Figure 1 of the main text, the box size was then expanded to a lower particle density of $\rho_N \simeq 0.4\,\sigma^{-3}$. At this reduced density, electrostatic repulsions between charged monomers are expected to play a more pronounced role, allowing the polymer chains to extend further than in the original sample. The system was equilibrated at this low density for $t = 10^5\tau$, after which it was instantaneous quenched to the density of the main sample under a constant-pressure simulation. This procedure effectively "freezes in" the extended chain (metastable) conformations achieved at low density, preserving the enhanced chain stiffening even after returning to the higher density conditions.

As a result, the end-to-end distance of the chains increases to $R_{\rm ee} \simeq 10.12\,\sigma$, approximately 6% larger than that of the main $\alpha = 84\%$ sample ($R_{\rm ee} \simeq 9.53\,\sigma$). Despite this further extension, the elastic modulus C_{11} remains essentially unchanged, indicating that the bulk stiffness of the system is not significantly affected. The thermal conductivity of this sample reaches $\kappa/\kappa(\alpha = 0\%) \simeq 3.1$, which is higher than in the main $\alpha = 84\%$ sample (i.e., $\kappa/\kappa(\alpha = 0\%) \simeq 2.5$). These observations provide strong evidence that chain stiffening induced by ionization is the major factor governing the increase in κ, whereas the mere presence of counterions alone plays a lesser role in enhancing heat transport.

[1] K. Kremer and G. S. Grest The Journal of Chemical Physics, vol. 92, no. 8, pp. 5057–5086, 1990.

[2] A. P. Thompson, H. M. Aktulga, R. Berger, D. S. Bolintineanu, W. M. Brown, P. S. Crozier, P. J. in't Veld, A. Kohlmeyer, S. G. Moore, T. D. Nguyen, et al., "Lammps-a flexible simulation tool for particle-based materials modeling at the atomic, meso, and continuum scales," Computer Physics Communications, vol. 271, p. 108171, 2022.

[3] S. Abdel-Azeim, "Revisiting opls-aa force field for the simulation of anionic surfactants in concentrated electrolyte solutions," Journal of Chemical Theory and Computation, vol. 16, no. 2, pp. 1136–1145, 2020. PMID: 31904948.

[4] J. Brandrup, E. H. Immergut, and E. A. Grulke, "Polymer handbook, 2 volumes set, 4th edition," 2003.

[5] A. Shanker, C. Li, G.-H. Kim, D. Gidley, K. P. Pipe, and J. Kim, "High thermal conductivity in electrostatically engineered amorphous polymers," Science Advances, vol. 3, no. 7, p. e1700342, 2017.

[6] J. Landsgesell, P. Hebbeker, O. Rud, R. Lunkad, P. Košovan, and C. Holm, "Grand-reaction method for simulations of ionization equilibria coupled to ion partitioning," Macromolecules, vol. 53, no. 8, pp. 3007–3020, 2020.

[7] R. Zwanzig, "Time-correlation functions and transport coefficients in statistical mechanics," 1965.
